# The association between long-term exposure to ambient PM_2.5_ and high-density lipoprotein cholesterol level among chinese middle-aged and older adults

**DOI:** 10.1186/s12872-024-03835-w

**Published:** 2024-03-21

**Authors:** Chaolin Liu, Yong Qiao

**Affiliations:** Department of surgery, Sichuan Province orthopedic hospital, Cheng, China

**Keywords:** HDL, PM_2.5_, Air pollution, China

## Abstract

**Background:**

Recently, the impact of PM_2.5_ on human health has been intensively studied, especially the respiratory system. High-density lipoprotein plays a crucial role in removing excess cholesterol from cells and transporting it to the liver for excretion. However, the effects of ambient PM2.5 on high-density lipoprotein (HDL) level have not been further studied. Our research aims to investigate the potential association between ambient PM_2.5_ concentrations and high-density lipoprotein (HDL) levels within the middle-aged and older adults in China.

**Methods:**

We employed data from individuals aged 45 years and above who were participants in Wave 3 of the China Health and Retirement Longitudinal Study (CHARLS). The high-quality, high-resolution PM_2.5_ exposure concentration data for each participant were obtained from the ChinaHighAirPollutants (CHAP) dataset, while the HDL levels were derived from blood samples collected during CHARLS Wave 3. This analysis constitutes a cross-sectional study involving a total of 12,519 participants. To investigate associations, we conducted multivariate linear regression analysis, supplemented by subgroup analysis.

**Results:**

In this cross-sectional investigation, we discerned a negative association between prolonged exposure to ambient PM_2.5_ constituents and high-density lipoprotein (HDL) levels. The observed correlation between ambient PM_2.5_ and HDL levels suggests that older individuals residing in areas with elevated PM_2.5_ concentrations exhibit a reduction in HDL levels (Beta: -0.045; 95% CI: -0.056, -0.035; *P* < 0.001). Upon adjusting for age in Model I, the Beta coefficient remained consistent at -0.046 (95% CI: -0.056, -0.035; *p* < 0.001). This association persisted even after accounting for various potential confounding factors (Beta = -0.031, 95% CI: -0.041, -0.021, *p* < 0.001).

**Conclusions:**

Our study reveals a statistically significant negative correlation between sustained exposure to higher concentrations of ambient PM_2.5_ and high-density lipoprotein (HDL) levels among Chinese middle-aged and older individuals.

**Supplementary Information:**

The online version contains supplementary material available at 10.1186/s12872-024-03835-w.

## Introduction

Dyslipidemia, a widely recognized and vital risk factor for cardiovascular disease, can lead to increases in global mortality and disease burdens [1]. Low levels of HDL cholesterol (high-density lipoprotein) are a common feature of dyslipidemia and are often associated with an increased risk of cardiovascular diseases, such as heart attacks and strokes [2]. HDL cholesterol is often referred to beneficial cholesterol because the main function of HDL is to transport cholesterol from the body’s tissues and cells to the liver for processing and excretion, which helps to prevent the buildup of excess cholesterol in the arteries and reduces the risk of cardiovascular diseases [3]. HDL also has anti-inflammatory and antioxidant properties, which can help protect the endothelial cells lining the blood vessels.

PM_2.5_ is a mixed particulate consisting of a mixture of solid particles and liquid droplets, which can contain a variety of chemical compounds including sulfates, nitrates, organic compounds, metals, and other pollutants [4]. Long-term exposure to concentrations of ambient PM2.5 is known to be associated with many health effects, including cardiopulmonary disorders, diabetes mellitus, and adverse health outcomes [5, 6]. Although existing researches have indicated that air pollution was significantly associated with dyslipidemia, there are some limitations [4, 7, 8]. The participants of Pan’s study [4] were limited to those who living in five provinces in southwest China and the research of Lei [8] only focused on essential hypertensive population. To the best of our knowledge, no study has yet examined the association between long-term ambient PM_2.5_ exposure and high-density lipoprotein (HDL) levels among adults at a national survey level. The potential influence of abnormal HDL level may be attributed to systemic inflammation or oxidative stress. Existing research has presented a limited number of possibilities, yet inadequate evidence is available to comprehensively elucidate the relationship between ambient PM2.5 and HDL levels.

Long-term exposure to ambient PM_2.5_ is known to be associated with many respiratory and circulatory diseases [6, 9, 10]. The HDL level, being a crucial physiological indicator among middle-aged and older individuals holds significant implications for their cardiovascular health, thereby impacting the economic and medical burdens borne by our society [11]. In a cohort study of 21 countries of various levels of income [9], a 10 µg increase in PM_2.5_ is associated with a 3% increase in the risk of death related to cardiovascular disease, a 5% increase in cardiovascular disease events, a 3% increase in myocardial infarction, and an 8% increase in stroke. As a crucial protective element against cardiovascular diseases, HDL plays a pivotal role in mitigating the accumulation of excessive cholesterol within the arteries and protecting the endothelial cells, which significantly reduces the incidence of cardiovascular diseases such as atherosclerosis. The findings of this study indicated that long-term exposure to air pollutants may cause a decrease in HDL levels. This revelation underscores the significance of governmental and societal initiatives aimed at regulating air pollutants. Furthermore, this adverse effect on HDL levels suggests a potential threat to cardiovascular health, particularly in the middle-aged and older adults. In light of the accelerating pace of population aging, it becomes imperative to advocate for sustainable and economically viable alternatives to conventional sources of pollution to safeguard the health of the middle-aged and older demographic.

## **Methods**

### Data Resource

The data utilized in this analysis were derived from the Wave 3 (2015) of the China Health and Retirement Longitudinal Study (CHARLS). This longitudinal study, conducted by the National School for Development at Peking University, is an ongoing investigation concentrating on the demographic and health dynamics of middle-aged and older populations in China. CHARLS provides longitudinal data on a variety of factors, such as socioeconomic status and health, in a nationally representative sample of middle-aged and older Chinese. The conceptual approach and metrics have been adapted to be consistent with the HRS and other sister surveys. CHARLS utilized the multistage stratified probability sampling method to obtain a representative sample of the population from 150 municipalities within 28 provinces, municipal communities, and autonomous regions. The CHARLS collected data on demographics, household characteristics, biomedical measurements, health status, and functioning for the first time in 2011–2012, and then every two years thereafter. The survey design has been described in detail elsewhere [12].

**Fig. 1:**
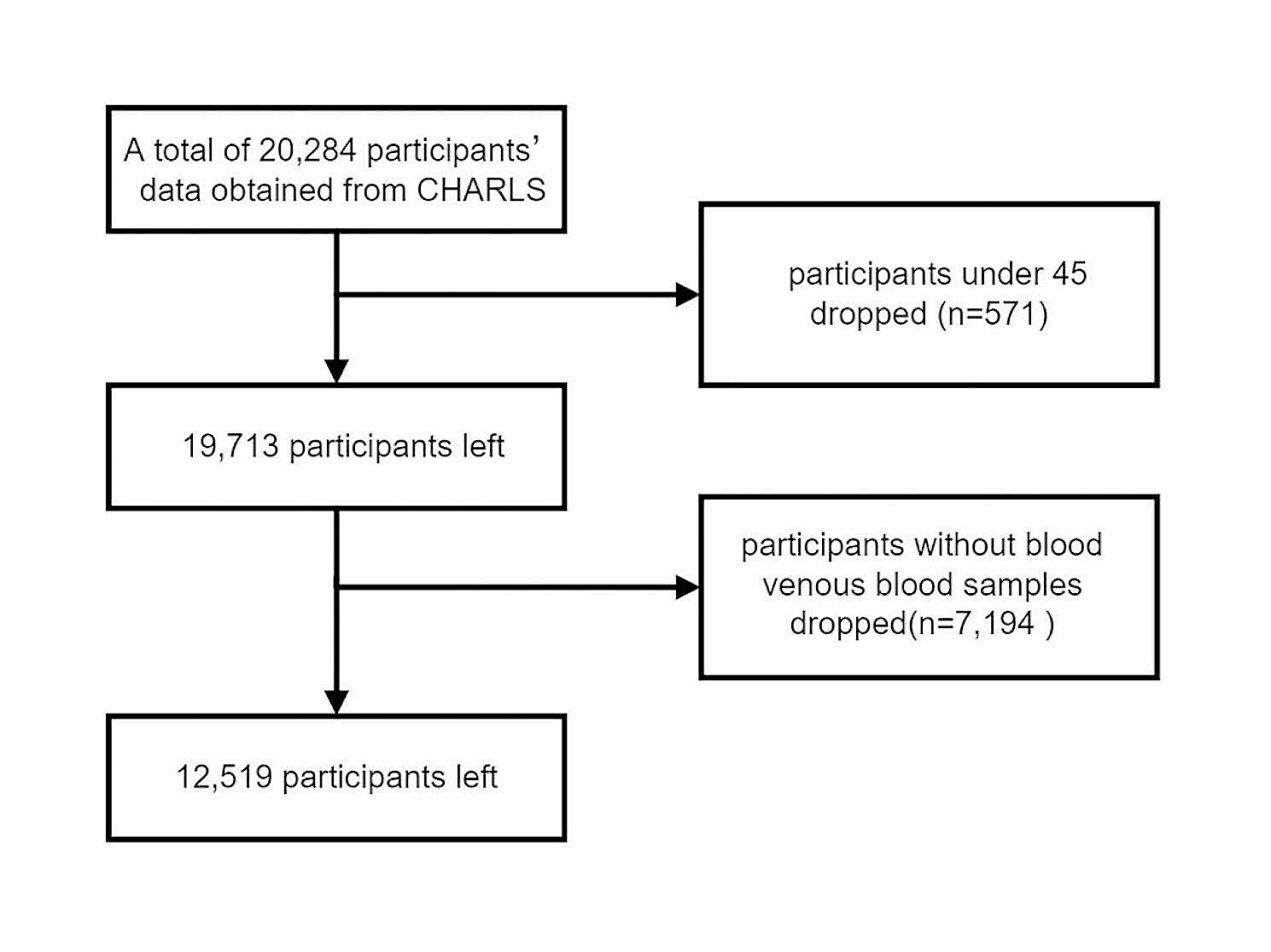
Flowchart of the participants selection

In wave 3, a total of 20,284 participants were questioned and asked to consent to venous blood samples. Participants under 45 years old (*n* = 571) and those without venous blood samples were excluded (*n* = 7,194). Eventually, a total of 12,519 adults were enrolled in our study. Further details on the inclusion and exclusion of study participants were shown in the Fig. 1.

### Definition of ambient PM_2.5_ and high-density lipoprotein cholesterol level

The high-quality and high-resolution data on PM_2.5_ exposure concentrations for each residential city were obtained from the CHAP dataset [13]. The CHAP dataset utilized a combination of multi-source satellite remote sensing and artificial intelligence technology, carefully considering the spatial and temporal heterogeneity of air pollution. This comprehensive approach integrated diverse datasets, including ground observations, satellite remote sensing products, atmospheric reanalysis, and model simulations, to generate high-quality PM_2.5_ data records at a 1-km resolution spanning the period from 2000 to 2018 in China. This dataset has been successfully employed in numerous studies investigating the impact of air pollution on public health, including examinations of associations with conditions such as diabetes and cardiovascular diseases [14, 15]. Given privacy considerations pertaining to individual residential addresses, access was limited to city-level PM_2.5_ data, serving as a proxy for individual-level data. Participants were consequently matched to the city-level average PM_2.5_ concentration. The PM_2.5_ exposure level for each individual was computed as the average from the inception of the study in 2011 to its conclusion in 2015.

The study targeted the entire cohort of 21,100 individuals who participated in the third wave of the China Health and Retirement Longitudinal Study (CHARLS). A response rate of 64% yielded a total of 13,420 collected blood samples. The analysis of blood samples occurred in two distinct phases. Initially, local county health centers promptly conducted a comprehensive blood count (CBC) analysis, encompassing hemoglobin, hematocrit, white blood cell count, platelet count, and mean corpuscular volume, immediately following blood collection. Subsequently, the samples were transported to the central study headquarters, where the measurement of high-density lipoprotein (HDL) cholesterol was carried out [16].

### Covariates

To address potential confounding variables, this study incorporated specific parameters known to exert influence on the HDL level. Age was quantified in units of years, education was categorized into three levels: less than lower secondary, upper secondary & vocational training, and tertiary. Marital status was dichotomized into two categories: married or partnered, and separated, divorced, widowed, or never married. Residential locations were differentiated as rural or urban. BMI was calculated by dividing the weight in kilograms by the square of the height in meters. Participants were stratified based on their history of smoking and drinking. Additionally, numerous chronic diseases were identified as potential confounding factors through a structured inquiry, asking participants, “Have you received a doctor’s diagnosis for any of the listed conditions?” These conditions encompassed hypertension, lung diseases, stroke, psychiatric disorders, arthritis, liver disease, kidney disease, stomach/digestive diseases, and asthma. Relying on predictive mean matching approaches, missing covariate variables were addressed using multivariate imputation techniques.

### Statistical analysis

Standard deviations and means are utilized as measures of central tendency and dispersion for continuous variables that adhere to a normal distribution in the baseline characteristics, while the interquartile range and median are employed to represent continuous variables that conform to a skewed distribution. Rates and percentages are used to depict categorical variables. The Kruskal-Wallis test was utilized to determine *p*-values for continuous variables conforming to a skewed distribution. Conversely, chi-square tests were employed to analyze categorical data. The Fisher exact test was applied in cases where the expected cell count was less than 10 [17].

The ambient PM_2.5_ and HDL levels were treated as continuous variables in this study. We stratified the baseline data by gender to examine whether there are potential disparities within the middle-aged and elderly populations. Multiple linear regression analysis was employed to explore the relationship between ambient PM_2.5_ and HDL levels. To mitigate the influence of potential confounding variables on the investigation’s outcomes, several covariates were adjusted. Participants were stratified into four age groups based on cutoffs of 50, 60, and 70. Model I was adjusted solely for age groups. Conversely, Model II incorporated adjustments for age, gender, education level, marital status, and residence. Furthermore, Model III included additional adjustments for smoking condition, drinking condition, and BMI. In the present study, Model IV encompassed further adjustments for various covariates, including hypertension, diabetes, lung diseases, heart diseases, stroke, psychological problems, arthritis, dyslipidemia, liver diseases, kidney diseases, digestive diseases, and asthma. To assess the heterogeneity of the association between ambient PM_2.5_ and HDL levels, interaction analyses were conducted, stratified by covariates. Subgroup analyses utilized stratified linear regression, and the *p*-value for interaction was determined through the log-likelihood ratio test, involving the comparison of models with and without covariate interactions. A significance level of 0.05 was deemed statistically significant for all the statistical findings in this study. The analyses were performed using R version 4.2.2.

## Results

### Baseline characteristics and the average level of PM_2.5_ concentration in China

**Fig. 2:**
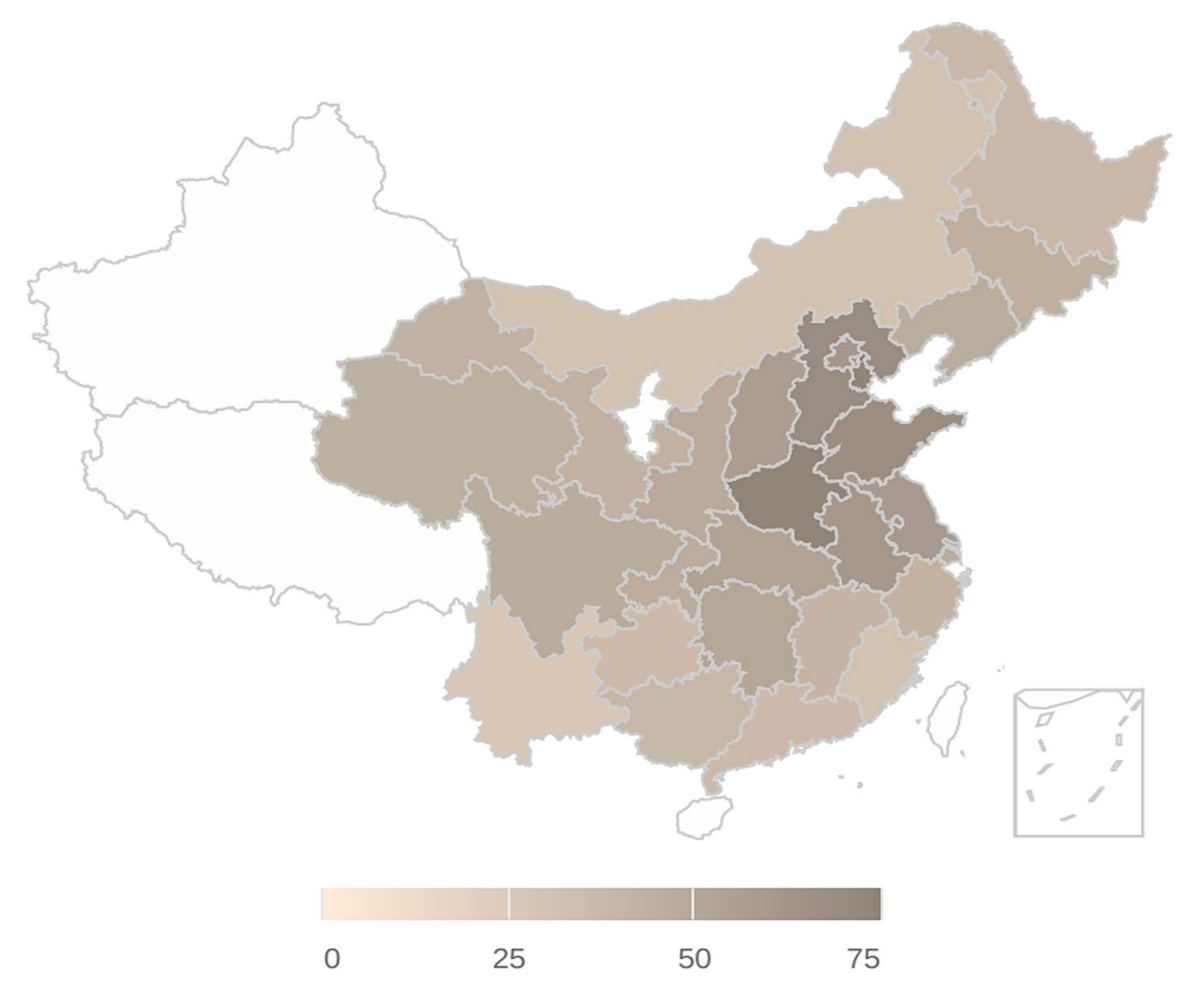
The annual average of PM_2.5_ concentration of participants living provinces

The study encompasses a cohort of 12,519 participants, and Table 1 presents the baseline characteristics of the individuals. Notably, at baseline, discernible evidence indicates that the female cohort exhibited higher levels of high-density lipoprotein (HDL) compared to their male counterparts (52.30 ± 10.80 mg/dl vs. 49.96 ± 12.38 mg/dl, *p* < 0.001). Simultaneously, an observation of note is that the female subgroup in our study demonstrated a lower mean age relative to the male subgroup (60.16 ± 9.74 vs. 61.33 ± 9.73). Furthermore, significant disparities (*P* < 0.001) were identified between the sexes concerning residence, educational attainment, marital status, alcohol consumption, tobacco use, and the prevalence of chronic diseases. Additionally, Fig. 2 illustrates the PM_2.5_ concentrations in the cities of residence for the study participants.

**Table 1:** Baselines characteristics of the participants enrolled (IQR: interquartile range; for continuous variables: *P* value was calculated by Kruskal Wallis rank-sum test, Number (%) for categorical variables: *P* value was calculated by chi-square test, HDL: high-density lipoprotein cholesterol)

		Gender		
		male	female	*P*
		*n* = 5889	*n* = 6630	
age (median [IQR])		61.00 [53.00, 68.00]	60.00 [52.00, 66.00]	< 0.001
age_class (%)	< 50	744 (12.63)	989 (14.92)	< 0.001
	< 60	1840 (31.24)	2283 (34.43)	
	< 70	2095 (35.57)	2194 (33.09)	
	>=70	1210 (20.55)	1164 (17.56)	
Residence (%)	urban	1163 (22.25)	1098 (18.51)	< 0.001
	rural	4064 (77.75)	4834 (81.49)	
Education level (%)	Less than lower secondary	5761 (97.86)	6571 (99.14)	< 0.001
	tertiary	126 (2.14)	57 (0.86)	
Marital status (%)	married or partnered	5293 (89.88)	5569 (84.00)	< 0.001
	separated divorced widowed or never married	596 (10.12)	1061 (16.00)	
Drinking frequence (%)	none	2473 (42.14)	5612 (84.95)	< 0.001
	less than once per day	1920 (32.72)	834 (12.62)	
	once per day	800 (13.63)	108 (1.63)	
	twice per day	490 (8.35)	45 (0.68)	
	more than twice per day	185 (3.15)	7 (0.11)	
Smoking frequence (%)	none	2750 (47.23)	6285 (95.14)	< 0.001
	<=4	293 (5.03)	58 (0.88)	
	<=10	763 (13.11)	139 (2.10)	
	> 10	2016 (34.63)	124 (1.88)	
hypertension (%)	no	3291 (65.56)	3664 (63.36)	0.018
	yes	1729 (34.44)	2119 (36.64)	
diabetes (%)	no	4543 (90.86)	5069 (88.34)	< 0.001
	yes	457 (9.14)	669 (11.66)	
Lung diseases (%)	no	4162 (82.63)	5107 (88.23)	< 0.001
	yes	875 (17.37)	681 (11.77)	
Heart diseases (%)	no	4195 (83.58)	4532 (78.80)	< 0.001
	yes	824 (16.42)	1219 (21.20)	
stroke (%)	no	4813 (95.51)	5621 (96.88)	< 0.001
	yes	226 (4.49)	181 (3.12)	
Psych problem (%)	no	4958 (98.28)	5637 (97.39)	0.002
	yes	87 (1.72)	151 (2.61)	
arthritis (%)	no	3072 (60.76)	2993 (51.93)	< 0.001
	yes	1984 (39.24)	2771 (48.07)	
dyslipidemia (%)	no	3974 (80.85)	4428 (78.80)	0.01
	yes	941 (19.15)	1191 (21.20)	
Liver diseases (%)	no	4638 (92.48)	5411 (93.99)	0.002
	yes	377 (7.52)	346 (6.01)	
Kidney diseases (%)	no	4455 (88.69)	5260 (91.27)	< 0.001
	yes	568 (11.31)	503 (8.73)	
Digest diseases (%)	no	3636 (71.97)	3792 (65.50)	< 0.001
	yes	1416 (28.03)	1997 (34.50)	
asthma (%)	no	4674 (92.59)	5484 (94.83)	< 0.001
	yes	374 (7.41)	299 (5.17)	
HDL (median [IQR])	mg/dl	48.26 [41.31, 56.37]	51.35 [44.79, 58.59]	< 0.001
BMI (median [IQR])	kg/m2	23.21 [20.88, 25.75]	24.13 [21.81, 26.69]	< 0.001
PM_2.5_ (median [IQR])		54.64 [39.90, 69.30]	54.64 [40.89, 69.44]	0.387

### Association between ambient PM_2.5_ and HDL level

The results of multivariate regression analyses investigating the relationship between ambient PM_2.5_ exposure and HDL levels among study participants are presented in Table 2. In the crude model, the analysis revealed a negative correlation between ambient PM_2.5_ and HDL levels, indicating that older individuals residing in areas with higher PM_2.5_ concentrations exhibited a decrease in HDL levels (Beta: -0.045; 95% CI: -0.056, -0.035; *P* < 0.001). Upon adjusting for age in Model I, the observed Beta remained significant at -0.046 (95% CI: -0.056, -0.035; *p* < 0.001). The association persisted in Model II (Beta: -0.047; 95% CI: -0.057, -0.036; *p* < 0.001) and Model III (Beta: -0.041; 95% CI: -0.051, -0.031, *p* < 0.001) even after accounting for potential confounding factors, such as smoking condition, drinking condition, and BMI. Model IV, which considered all variables, continued to demonstrate statistically significant findings (Beta = -0.031, 95% CI: -0.041, -0.021, *p* < 0.001). The analysis based on Model I suggests that age potentially plays a role in influencing the association between ambient PM_2.5_ and HDL levels. Model III further implies that variables such as smoking condition, drinking condition, and BMI may collectively contribute to this association. Lastly, the findings of Model IV underscore that the presence of comorbidities significantly influences the overall observed correlation.

**Table 2:** Multivariate regression model of the relationship between PM_2.5_ and the level of HDL Multivariate liner regression was used to identify the association between ambient PM_2.5_ and HDL level ; CI: confidence interval; BMI: body mass index

Exposure	Crude model	
	Beta (95%CI)	*P*-value
Crude model	-0.045(-0.056, -0.035)	<0.001
Model I	-0.046(-0.056, -0.035)	<0.001
Model II	-0.047(-0.057, -0.036)	<0.001
Model III	-0.041(-0.051, -0.031)	<0.001
Model IV	-0.031(-0.041, -0.021)	<0.001

Crude model adjust for none;

Model I adjust for: age

Model II adjust for: age; gender; education level; marital status; residence

Model III adjust for: age; gender; education level; marital status; residence; smoking condition; drinking condition; BMI

Model IV adjust for: age; gender; education level; marital status; residence; smoking condition; drinking condition; BMI; hypertension; diabetes; lung diseases; heart diseases; stroke; psych problems; arthritis; dyslipidemia; liver diseases; kidney diseases; digest diseases; asthma

### Subgroup analysis and sensitivity analysis

**Fig. 3:**
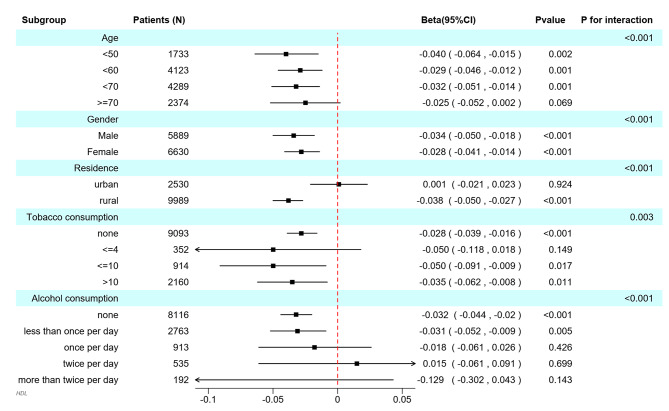
Subgroup analysis between ambient PM_2.5_ and high-density lipoprotein cholesterol level; 95% CI: 95% Confidence interval;

The results depicted in Fig. 3 reveal a noteworthy association between ambient PM_2.5_ levels and high-density lipoprotein cholesterol among individuals segmented by age and gender. Moreover, this observed correlation persists consistently across diverse demographic strata, encompassing residents of rural areas. Importantly, this consistency is sustained for individuals reporting both non-smoking habits or a daily cigarette consumption exceeding four, as well as those reporting either non-drinking habits or a frequency of alcohol consumption less than once per day. Furthermore, the outcomes of the interaction analysis indicate a statistically significant impact of variables such as age, gender, residential status, tobacco use, and alcohol consumption. We systematically organized the initial dataset based on gender and subsequently conducted distinct multiple linear regression analyses for middle-aged and elderly individuals, distinguishing between men and women (Supplementary Table S1). In the meantime, we also adjusted the models for age, education, smoking, alcohol consumption, and chronic diseases according to the main analysis, which exhibited concordance with the main analytical findings.

## Discussion

In light of a pronounced aging demographic, China is currently undergoing substantial population growth, with a conspicuous escalation anticipated in the proportion of individuals aged 60 and above from 2001 to 2031 [18]. Simultaneously, as the nation undergoes continual growth, the expansion of industrial activities and the consequential air pollution such as PM_2.5_ pose threats to public health. As a crucial diagnostic parameter for cardiovascular and cerebrovascular diseases, HDL assumes a pivotal role in attenuating the accumulation of excessive cholesterol within arterial walls and safeguarding endothelial cells. This investigation represents one of the pioneering studies dedicated to elucidating the association between prolonged exposure to ambient PM_2.5_ and the HDL level among middle-aged and order adults.

As a cross-sectional study, the present research discerned a negative correlation between extended exposure to ambient PM_2.5_ constituents and HDL levels. The observed relationship between ambient PM_2.5_ and HDL levels revealed that older individuals residing in areas with higher concentrations of PM_2.5_ exhibited a diminished level of HDL (Beta: -0.045; 95% CI: -0.056, -0.035; *P* < 0.001). Notably, this association persisted even after accounting for various potential confounding factors (Beta = -0.031, 95% CI: -0.041, -0.021, *p* < 0.001). This adverse impact of PM2.5 on HDL level remained consistently significant across all the models and the finding aligns cohesively with the overarching hypothesis posited in our study. Furthermore, within the gender-stratified analysis, PM2.5 exhibited a more significant influence on HDL levels among older men compared to the older women, which may be attributed to the differences in hormone and metabolic levels caused by gender.

Numerous previous investigations have explored the deleterious impacts of PM_2.5_ on overall health status and psychological well-being [19–21]. The extant literature consistently demonstrates a noteworthy association between air pollutants and dyslipidemia. Nevertheless, there is a scarcity of investigations specifically delving into the relationship between ambient PM_2.5_ exposure and high-density lipoprotein (HDL) level. While several antecedent studies have delineated a correlation between these variables [4, 8, 22, 23], the generalizability of their findings is impeded by certain methodological limitations and drawbacks. The study of Pan investigated the association of long-term exposure to ambient PM_2.5_ and dyslipidemia in 67,015 participants from the China Multi-Ethnic Cohort study [4]. Although they investigated the correlation between PM_2.5_ and dyslipidemia symptoms, the further relationship between HDL level and PM_2.5_ concentration was not explored. Meanwhile, the study’s participants were limited to those who living in five provinces in southwest China. Lei [8] found that PM_2.5_ and chemical constituents were associated with elevated levels of TC and TG in 37,530 participants with essential hypertension. The study of Rajkumar investigated the effect of household air pollution exposure on cardiovascular disease risk factors in 150 women in rural Honduras and observed a suggestive effect between metabolic syndrome and exposure to household air pollution. However, both studies mentioned above were only conducted on specific populations. While the comprehensive understanding of the potential biological mechanisms underpinning the correlation between PM_2.5_ constituents and dyslipidemia remains elusive, extant toxicological and epidemiological investigations propose that exposure to PM_2.5_ or its components may elicit systemic inflammation or provoke oxidative stress, thereby potentially leading to aberrations in HDL levels [4, 24, 25]. Several studies have posited the potential translocation of air pollutants across the alveolar barrier into the circulatory system, thereby contributing to oxidative stress and systemic inflammation, which may finally induce metabolic disruption [26, 27]. Also, the study of Li [28] indicated that air pollutants might induce disturbances in circadian rhythms first, and then lead to abnormal lipid metabolism in the liver. While previous studies have elucidated some potential mechanisms linking PM_2.5_ to HDL, further experimental studies are needed establish definitive associations between PM_2.5_ and HDL.

Although this research was conducted based on a nationwide investigation, it did have a few limitations. Firstly, our study was cross-sectional designed. The authority of the causal associations of PM2.5 with HDL and its components is weak. Further longitudinal studies are demanded to estimate the effects of PM2.5 on HDL level. Secondly, the accuracy of the survey results may be affected by recall bias due to the questionnaire survey and the elderly subjects. Thirdly, the inclusion of covariates remains insufficient. Some confounding factors were not considered, such as green space, noise and other factors. Fourthly, air pollution exposure of participants was based on community location, which can’t completely represent individual air pollutants exposure. Finally, our study was conducted among the Chinese adults. Therefore, the results should be extrapolated with caution.

## Conclusions

Our study reveals a statistically significant negative correlation between sustained exposure to higher concentrations of ambient PM_2.5_ and high-density lipoprotein (HDL) levels among Chinese middle-aged and older individuals. Notably, even after accounting for all potential covariates, the concentration of ambient PM_2.5_ was found to be causally linked to a decline in HDL levels. In light of the accelerating pace of population aging, it becomes imperative to advocate for sustainable and economically viable alternatives to conventional sources of pollution, in order to safeguard the health of the middle-aged and older demographic. Furthermore, additional prospective investigations are imperative to elucidate the aforementioned correlation and delve into potential underlying mechanisms.

### Electronic supplementary material

Below is the link to the electronic supplementary material.

Supplementary Material 1

## Data Availability

Publicly available datasets were analyzed in this study, which can be found at: CHARLS website (https://charls.pku.edu.cn/) and CHAP website (https://weijing-rs.github.io/index.html).
