# Supplementary material for: The association between long-term exposure to ambient PM2.5 and high-density lipoprotein cholesterol level among chinese middle-aged and older adults
Source: BMC Cardiovasc Disord. 2024 Mar 21;24:173. doi: 10.1186/s12872-024-03835-w (PMC10956307; doi:10.1186/s12872-024-03835-w)
Supplement: Supplementary file 1 — Supplementary Material 1 [file 12872_2024_3835_MOESM1_ESM.docx]

***Supplementary Material***

**The association between long-term exposure to ambient PM2.5 and high-density lipoprotein cholesterol level among Chinese older adults**


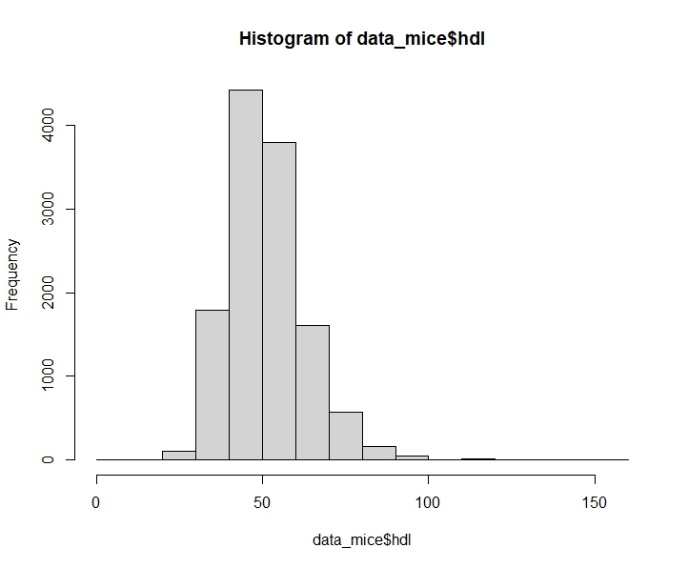


Supplementary figure1 of HDL Distribution


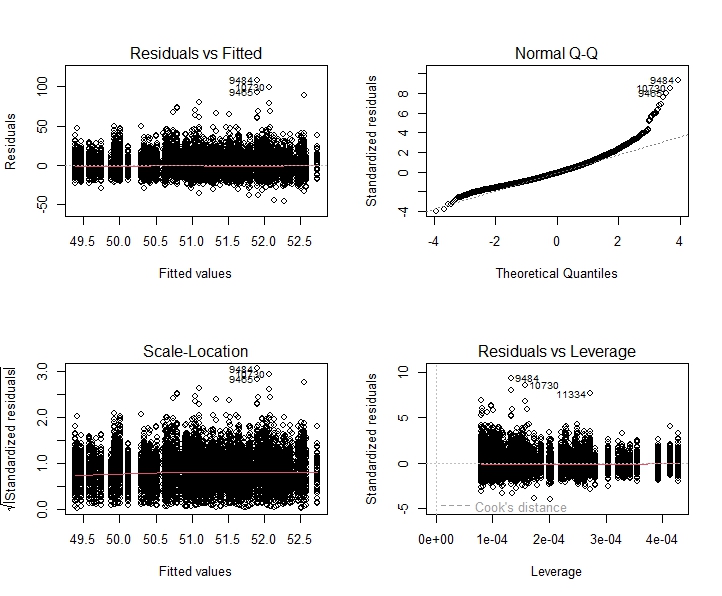


Supplementary figure2 of residual analysis

| **Supplement Table S1 Multivariate regression model of the relationship between PM2.5 and the level of HDL between men and women--Sensitivity analysis** | | | | |
| --- | --- | --- | --- | --- |
| Exposure | Male | | Female | |
|  | Beta (95%CI) | P-value | Beta (95%CI) | P-value |
| Crude model | -0.053(-0.07,-0.037) | <0.0001 | -0.039(-0.053,-0.026) | <0.0001 |
| Model I | -0.053(-0.07,-0.037) | <0.0001 | -0.04(-0.053,-0.026) | <0.0001 |
| Model II | -0.053(-0.07,-0.037) | <0.0001 | -0.04(-0.053,-0.026) | <0.0001 |
| Model III | -0.045(-0.061,-0.03) | <0.0001 | -0.037(-0.051,-0.024) | <0.0001 |
| Model IV | -0.034(-0.05,-0.018) | <0.0001 | -0.028(-0.041,-0.014) | <0.0001 |
| Crude model adjust for none； | | | | |
| Model I adjust for: age | | | | |
| Model II adjust for: age; education level; marital status; residence | | | | |
| Model III adjust for: age; education level; marital status; residence; smoking condition; drinking condition; BMI | | | | |
| Model IV adjust for: age; education level; marital status; residence; smoking condition; drinking condition; BMI; hypertension; diabetes; lung diseases; heart diseases; stroke; psych problems; arthritis; dyslipidemia; liver diseases; kidney diseases; digest diseases; asthma | | | | |

**Supplement Table S1. Sensitivity analysis and BH test for prostatitis (fix effect model)**
